# Synthesis, characterization and olefin polymerization behaviors of phenylene-bridged bis-β-carbonylenamine binuclear titanium complexes†

**DOI:** 10.1039/c8ra00071a

**Published:** 2018-02-13

**Authors:** Derong Luo, Yi Zeng, Xiong Chen, Ping Xia, Guangyong Xie, Qingliang You, Li Zhang, Tingcheng Li, Xiangdan Li, Aiqing Zhang

**Affiliations:** Key Laboratory of Catalysis and Materials Science of the State Ethnic Affairs Commission, Ministry of Education Hubei Province, South-Central University for Nationalities Wuhan 430074 China xiegy@scuec.edu.cn; Key Laboratory of Optoelectronic Chemical Materials and Devices, Ministry of Education, School of Chemical and Environmental Engineering, Jianghan University Wuhan 430056 China

## Abstract

Binuclear and multinuclear complexes have attracted much attention due to their unique catalytic performances for olefin polymerization compared with their mononuclear counterparts. In this work, a series of phenyl-bridged bis-β-carbonylenamine [O^−^NS^R^] (R = alkyl or phenyl) tridentate ligands and their binuclear titanium complexes (Ti^2^L_1_–Ti^2^L_5_) were synthesized and characterized by ^1^H NMR, ^13^C NMR, FTIR and elemental analysis. The molecular structure of ligand L_2_ (R = ^*n*^Pr) and its corresponding Ti complex Ti^2^L_2_ were further investigated by single-crystal X-ray diffraction, which showed that each titanium coordinated with six atoms to form a distorted octahedral configuration along with the conversion of the ligand from β-carbonylenamine to β-imino enol form. Under the activation of MMAO, these complexes catalyzed ethylene polymerization and ethylene/α-olefin copolymerization with extremely high activity (over 10^6^ g mol (Ti)^−1^ h^−1^ atm^−1^) to produce high molecular weight polyethylene. At the same time, wider polydispersity as compared with the mononuclear counterpart TiL_6_ was observed, indicating that two active catalytic centers may be present, consistent with the asymmetrical crystal structure of the binuclear titanium complex. Furthermore, these complexes possessed better thermal stability than their mononuclear analogues. Compared with the complexes bearing alkylthio sidearms, the complex Ti^2^L_5_ bearing a phenylthio sidearm exhibited higher catalytic activity towards ethylene polymerization and produced polyethylene with much higher molecular weight, but with an appreciably lower 1-hexene incorporation ratio. Nevertheless, these bis-β-carbonylenamine-derived binuclear titanium complexes showed much higher ethylene/1-hexene copolymerization activity and 1-hexene incorporation ratios as compared with the methylene-bridged bis-salicylaldiminato binuclear titanium complexes, and the molecular weight and 1-hexene incorporation ratio could be flexibly tuned by the initial feed of α-olefin commoners and catalyst structures.

## Abstract 2

Phenyl-bridged bis-β-carbonylenamine binuclear titanium complexes were synthesized, characterized and used to catalyze ethylene (co)polymerization with extremely high activity.

## Graphical abstract

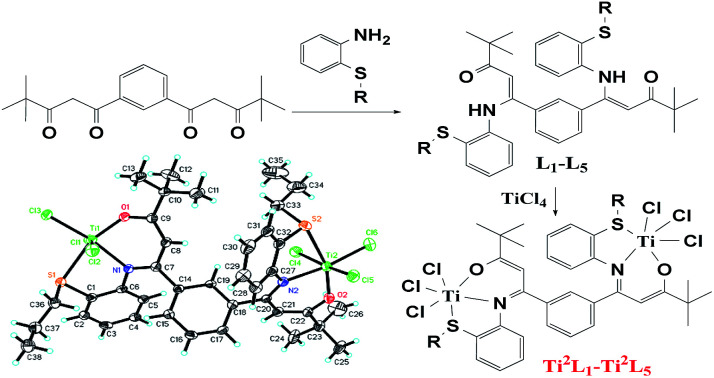

## 1 Introduction

Polyolefins are by far the most important and most produced synthetic polymers today, and the design and synthesis of effective catalysts for olefin polymerization and copolymerization is of great interest in both academic research and industrial applications. The discovery of single-site group 4 metallocene catalysts is considered one of the most significant breakthroughs after the discovery of Ziegler-Natta catalysts.^1^ Thereafter, single-site non-metallocene catalytic systems, including early and late transition metals catalysts, are thought of as another significant breakthrough, since they can provide novel olefin-based materials with superior activity and greater control over polymer microstructures.^2,3^

**Chart 1:**
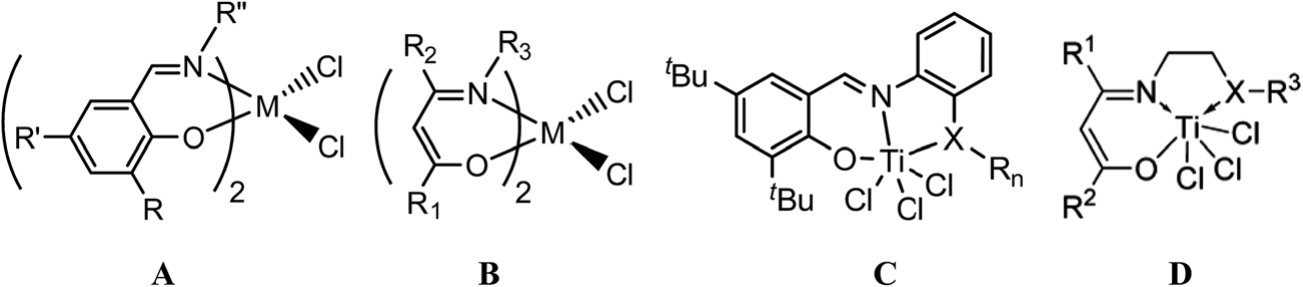
Some typical mononuclear titanium complexes.

Of the non-metallocene candidates, the group 4 non-metallocene complexes with bidentate anionic [N, O] chelate ligands, which was first reported in 1995,^4^ have been the focus of attention. A great variety of [N, O] chelate complexes have been reported, among which the most prominent were the group 4 bis(phenoxyimine) ligated complexes (A, Chart 1) reported firstly by Floriani *et al.* in 1995.^5^ Fujita *et al.*^6^ and Coates *et al.*^7^ further developed these ligands and reported some new complexes that are excellent for olefin polymerization including ethylene living polymerization, highly syndiospecific propylene living polymerization, living copolymerization of ethylene with α-olefin, and the synthesis of functional and block copolymers of propylene. However, very few successful phenoxyimine catalysts have been reported that effectively catalyze random copolymerization of ethylene and other olefins due to the low comonomer incorporation ratio. The group 4 transition metal complexes based on bis(β-carbonylenamine) ligands (B, Chart 1) were another group of widely-researched ethylene (co)polymerization precatalysts containing [N, O] chelate ligands.^4,8^

To further improve the catalytic performances of the bidentate anionic [N, O] chelated complexes towards ethylene (co)polymerization, Tang and coworkers have developed a series of mono-ligated tridentate [ONX]TiCl_3_ complexes (X = O, S, Se, and P) based on either salicylaldiminato or β-carbonylenamine backbone by introducing some sidearms with pendant-coordination heteroatom groups (C and D, Chart 1),^9^ which exhibited better catalytic performances due to the tuning of the electronic and steric properties of the active species by the sidearm. These complexes were especially effective for ethylene copolymerization with α-olefins, cycloolefins or polar monomers, due partially to the less crowded coordination sphere. Furthermore, they could be prepared in one step by simply mixing the tridentate ligands and titanium tetrachloride, without the need to deprotonate the ligands in advance.

**Chart 2:**
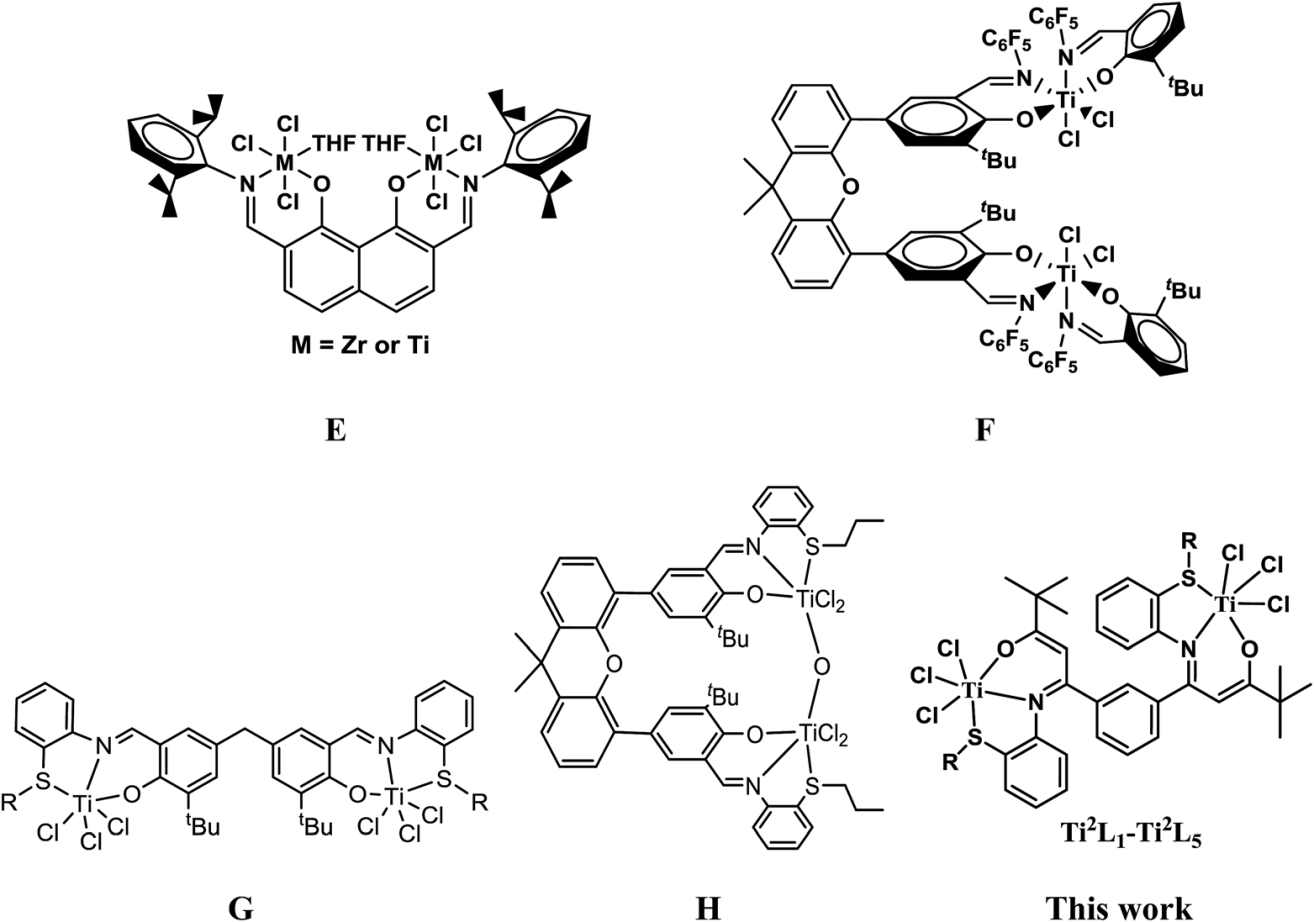
Non-metallocene binuclear titanium complexes.

More recently, there have also been growing interests in bi- and multi-nuclear olefin polymerization catalysts,^3*a*–*d*,10–13^ which showed that introducing a proximate metal center could significantly enhance catalytic properties as compared with the mononuclear analogue due to the creation of high local reagent concentrations, conformationally advantageous active-site-substrate proximities, as well as multicenter directed covalent and noncovalent interactions. Most of these works were focused on metallocene and late transition metal complexes, while early transition non-metallocene binuclear complexes have rarely been reported. Marks' group reported a class of naphthoylimine-ligated early transition bimetallic catalysts (E, Chart 2) with moderate activity (∼10^4^ g mol^−1^ h^−1^ atm^−1^) and higher comonomer incorporation ratios compared to the mononuclear analogue due to nuclearity and cooperativity effects in binuclear catalysts.^10*c*,*d*^ Ma and coworkers lately described a bidentate salicylaldimine heteroligated binuclear titanium catalyst (F, Chart 2) with high activity and higher ethylene/1,5-hexadiene copolymerization capability than that of its mononuclear counterpart.^13^

Recently we have been committed to developing some novel early and late transition non-metallocene catalysts based on tuning the coordination environment of the active species with electronic and/or steric effects of the substituents.^14^ In view of the advantage of the less crowded coordination sphere of tridentate ligands and the cooperative effect of binuclear complexes, we have designed a number of binuclear titanium catalysts with methylene- or xanthene-bridged bis(salicylaldiminato) tridentate ligands (G and H, Chart 2) and investigated their catalytic behaviors for ethylene homo- and copolymerization.^15^ Here we describe the synthesis, structure and ethylene (co)polymerization behaviors of a series of novel phenyl-bridged bis-β-carbonylenamine [O^−^NS^R^] (R = alkyl or phenyl) tridentate binuclear titanium complexes Ti^2^L_1_–Ti^2^L_5_ (Chart 2).

## 2 Results and discussion

### 2.1. Synthesis and structure of ligands and binuclear Ti complexes

**Scheme 1:**
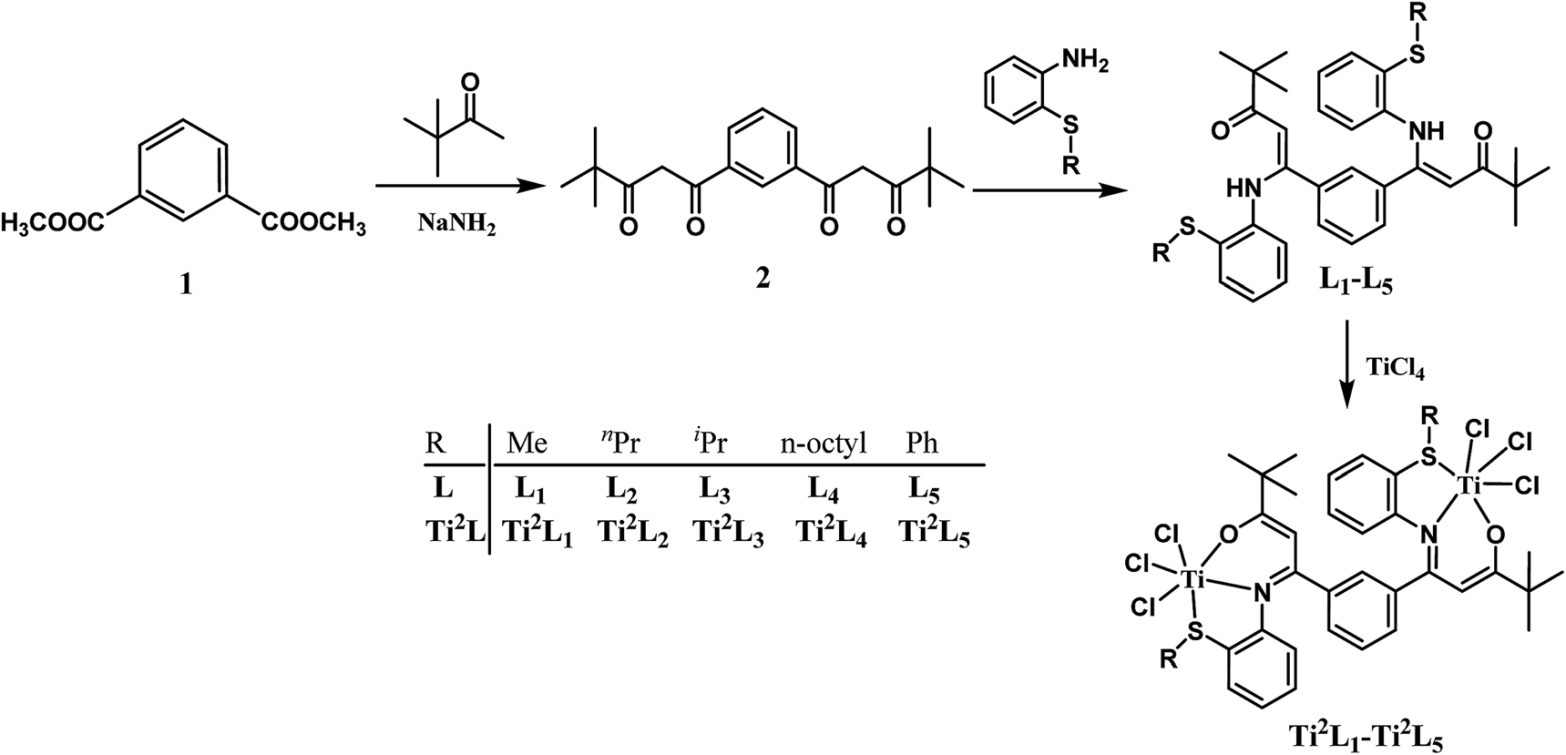
Synthesis of binuclear Ti complexes Ti^2^L_1_–Ti^2^L_5_.

**Scheme 2:**
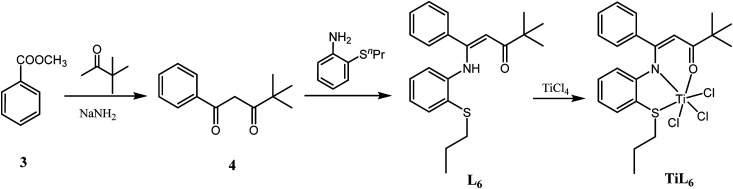
Synthesis of mononuclear Ti complex TiL_6_.

The synthetic routes for the ligands L_1_–L_6_ and the corresponding complexes Ti^2^L_1_–Ti^2^L_5_ and TiL_6_ were shown in Scheme 1 and 2.

Firstly, 1,1′-(1,3-phenylene)-bis(4,4-dimethylpentane-1,3-dione) (2) and 4,4-dimethyl-1-phenylpentane-1,3-dione (4) were synthesized according to the work of L. F. Lindoy's group^16^ through Claisen condensation of dimethyl isophthalate or methyl benzoate with pinacolone deprotonated by sodium amide, which then reacted with alkylthio anilines to obtain bis- and mono-β-carbonylenamine [ONS] tridentate ligands L_1_–L_6_ in 68–83% yields. Finally, binuclear Ti complexes Ti^2^L_1_–Ti^2^L_5_ and mononuclear counterpart TiL_6_ were prepared by reacting the ligands L_1_–L_6_ directly with excess TiCl_4_ according to Tang's^9^ and our previous works.^15^ The structures of the free ligands and the corresponding bi- and mono-nuclear Ti complexes were characterized by ^1^H NMR, ^13^C NMR, FT IR and elemental analysis. Notable changes of the ^1^H NMR spectra were that the NH resonances of the ligands at *δ* 12.05–12.22 ppm disappeared upon forming the Ti complexes.

**Fig. 1:**
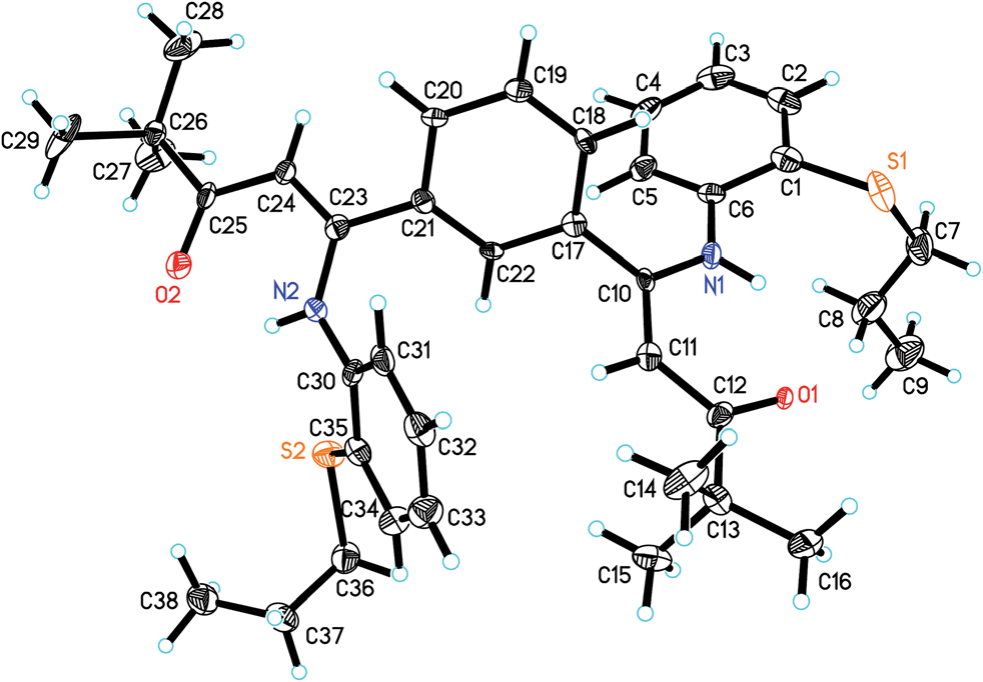
The crystal structure of ligand L_2_.

**Fig. 2:**
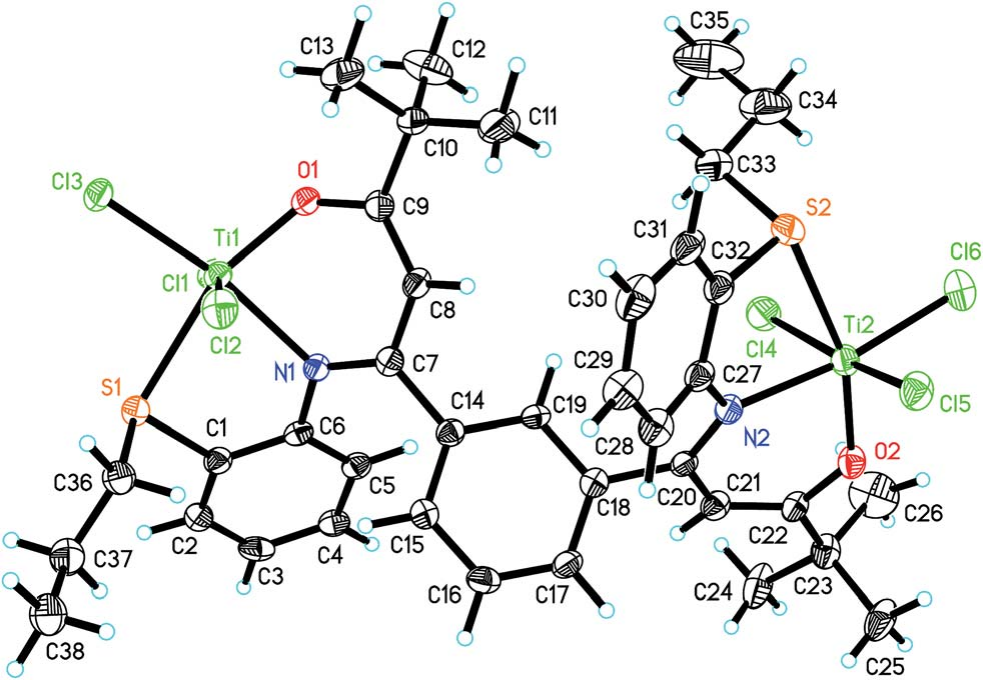
The crystal structure of complex Ti^2^L_2_.

The molecular structures of ligand L_2_ and its corresponding Ti complex Ti^2^L_2_ were further confirmed by single-crystal X-ray diffraction, as shown in Fig. 1 and 2. The crystal data and details of data collection and refinement are summarized in Table 1, and selected bond lengths and angles are listed in Table 2.

**Table 1:** The crystal data and structure refinement for ligand L_2_ and complex Ti^2^L_2_

	L_2_	Ti^2^L_2_
Empirical formula	C_38_H_48_N_2_O_2_S_2_	C_38_H_46_Cl_6_N_2_O_2_S_2_Ti_2_
Formula weight	628.90	935.39
Crystal size (mm^3^)	0.08 × 0.05 × 0.03	0.211 × 0.165 × 0.112
Crystal system	Monoclinic	Monoclinic
Space group	*P*12_1_/*c*1	*P*2_1_/*c*
*a* (Å)	14.149(5)	18.572(3)
*b* (Å)	10.258(3)	14.833(3)
*c* (Å)	24.194(8)	18.032(3)
*α* (°)	90°	90°
*β* (°)	96.710(6)°	108.118(3)°
*γ* (°)	90°	90°
*V* (Å^3^)	3487.7(19)	4721.4(14)
*Z*	4	4
Density (Mg m^−3^)	1.198	1.316
Absorption coefficient (mm^−1^)	0.188	0.798
*θ*_max_/°	25.248°	25.5°
Reflections collected/unique	22 094/6060 [*R*(int) = 0.1731]	31 859/8776 [*R*(int) = 0.0680]
Goodness-of-fit on *F*^2^	0.963	1.002
Final *R* indices [*I* > 2*σ*(*I*)]	*R*_1_ = 0.0916, w*R*_2_ = 0.2043	*R*_1_ = 0.0705, w*R*_2_ = 0.1858

**Table 2:** Selected bond lengths (Å) and bond angles (°) for ligand L_2_ and complex Ti^2^L_2_

L_2_		Ti^2^L_2_			
S(1)–C(1)	1.769(7)	S(1)–C(1)	1.780(5)	O(1)–Ti(1)–N(1)	84.26(15)
S(1)–C(7)	1.845(8)	S(1)–C(36)	1.819(6)	O(1)–Ti(1)–Cl(3)	104.94(12)
S(2)–C(35)	1.772(6)	S(2)–C(32)	1.755(6)	N(1)–Ti(1)–Cl(3)	170.77(12)
S(2)–C(36)	1.831(7)	S(2)–C(33)	1.826(6)	O(1)–Ti(1)–Cl(2)	92.15(13)
O(1)–C(12)	1.251(7)	O(1)–C(9)	1.338(6)	N(1)–Ti(1)–Cl(2)	86.38(12)
O(2)–C(25)	1.225(7)	O(2)–C(22)	1.322(6)	Cl(3)–Ti(1)–Cl(2)	92.34(6)
N(1)–C(10)	1.376(7)	N(1)–C(7)	1.315(6)	O(1)–Ti(1)–Cl(1)	96.64(13)
N(2)–C(23)	1.370(7)	N(2)–C(20)	1.302(6)	N(1)–Ti(1)–Cl(1)	86.44(12)
C(10)–C(11)	1.400(8)	C(7)–C(8)	1.447(7)	Cl(3)–Ti(1)–Cl(1)	93.22(6)
C(11)–C(12)	1.444(8)	C(8)–C(9)	1.351(7)	Cl(2)–Ti(1)–Cl(1)	168.04(7)
C(23)–C(24)	1.379(8)	C(20)–C(21)	1.447(7)	O(1)–Ti(1)–S(1)	161.35(12)
C(24)–C(25)	1.441(8)	C(21)–C(22)	1.367(7)	N(1)–Ti(1)–S(1)	77.11(11)
		Ti(1)–O(1)	1.825(3)	Cl(3)–Ti(1)–S(1)	93.71(6)
		Ti(1)–N(1)	2.172(4)	Cl(2)–Ti(1)–S(1)	87.51(6)
		Ti(1)–Cl(3)	2.2599(16)	Cl(1)–Ti(1)–S(1)	81.57(6)
		Ti(1)–Cl(2)	2.2941(17)	O(2)–Ti(2)–N(2)	84.69(16)
		Ti(1)–Cl(1)	2.2997(16)	O(2)–Ti(2)–Cl(6)	105.11(12)
		Ti(1)–S(1)	2.5642(16)	N(2)–Ti(2)–Cl(6)	170.20(13)
				O(2)–Ti(2)–Cl(5)	99.81(13)
				N(2)–Ti(2)–Cl(5)	87.78(12)
				Cl(6)–Ti(2)–Cl(5)	90.35(7)
				O(2)–Ti(2)–Cl(4)	91.01(13)
				N(2)–Ti(2)–Cl(4)	86.13(12)
				Cl(6)–Ti(2)–Cl(4)	93.72(7)
				Cl(5)–Ti(2)–Cl(4)	167.04(7)
				O(2)–Ti(2)–S(2)	161.71(12)
				N(2)–Ti(2)–S(2)	77.18(12)
				Cl(6)–Ti(2)–S(2)	93.03(6)
				Cl(5)–Ti(2)–S(2)	82.01(6)
				Cl(4)–Ti(2)–S(2)	85.48(7)

**Scheme 3:**
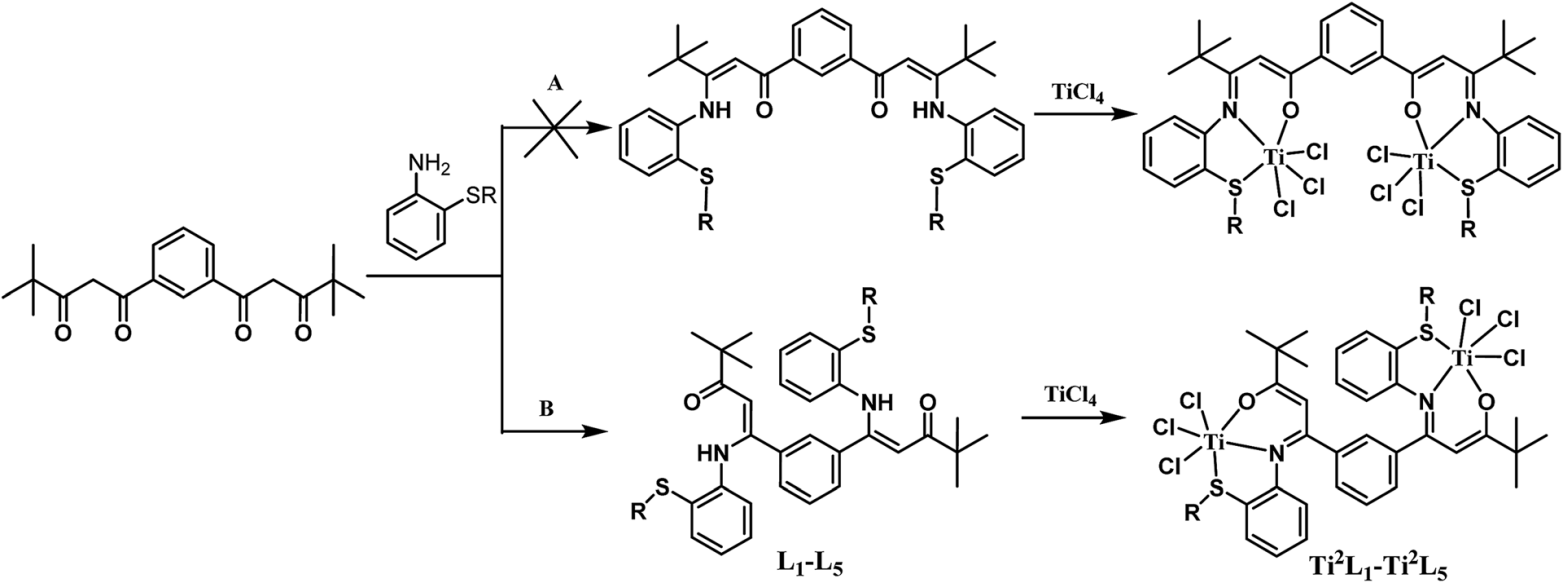
Two possible pathways for the synthesis of ligands L_1_–L_5_ and complexes Ti^2^L_1_–Ti^2^L_5_.

**Fig. 3:**
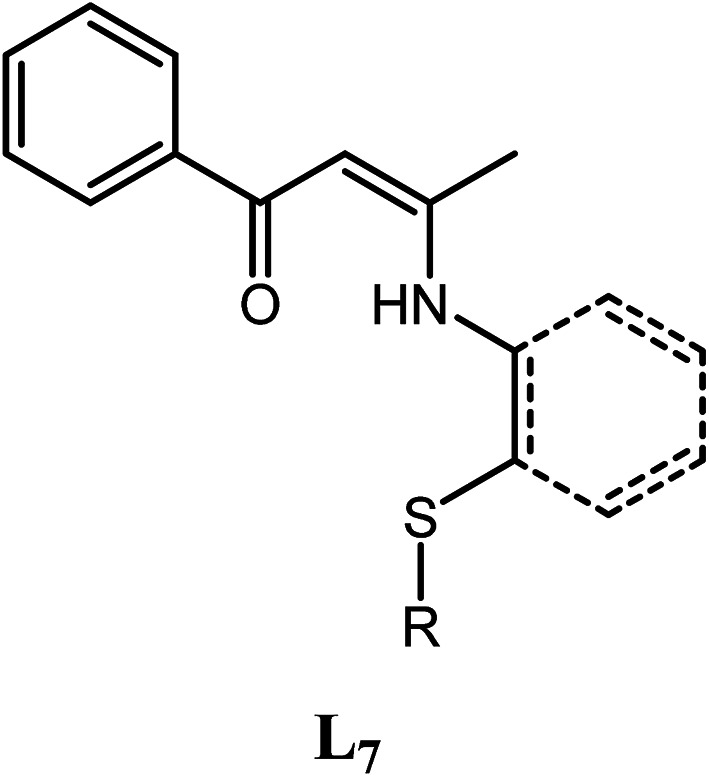
Mono-β-carbonylenamine ligand L_7_ derived from 1-phenylbutane-1,3-dione.

Two possible pathways existed for the synthesis of ligands L_1_–L_5_ and complexes Ti^2^L_1_–Ti^2^L_5_ due to the presence of two different carbonyl groups in phenylene-bridged β-dione (2), as shown in Scheme 3. In the case that 1-phenylbutane-1,3-dione was employed for the preparation of enamine, X-ray crystallographic analysis showed that the acetyl group of 1-phenylbutane-1,3-dione reacted with amine (L_7_, Fig. 3).^9*c*^ However, in the case of our bis-β-carbonylenamine ligands L_1_–L_5_, the single-crystal XRD proved that the alkylthio anilines reacted with the carbonyl group adjacent to phenylene group (Path B), not the one next to the ^*t*^butyl group (Path A), which resulted in far-separated and relatively independent titanium centers in complexes Ti^2^L_1_–Ti^2^L_5_ and would profoundly influence their catalytic performances for ethylene (co)polymerization.

From Fig. 1, it can be seen that in L_2_, the N1–C10–C11–C12–O1 and N2–C23–C24–C25–O2 are almost coplanar, which further formed two six-member rings *via* intramolecular hydrogen bonds between H and O1 or O2. The O1–C12 and O2–C25 bond lengths are 1.251 and 1.255 Å, respectively, a little longer than the typical C=O double bond but much shorter than C–O single bond. The C10–N1 and C23–N2 bond distances are 1.376(7) and 1.370(7) Å, respectively, showing clearly that the C–N bonds are single bonds. Thus, the ligand L_2_ exists in β-carbonylenamine form. The C11–C12 and C24–C25 bond lengths are 1.444(8) and 1.441(8) Å, respectively, and the C10–C11 and C23–C24 bond lengths are 1.400(8) and 1.379(8) Å, respectively, which are all between C–C single bond (1.54 Å) and C=C double bond (1.34 Å) and show a certain extent of delocalization of the double bonds. Furthermore, the distances of the corresponding bonds in two β-carbonylenamine units are a little different, which would provide different coordination environments for the two titanium metal centers.

As revealed by XRD analysis, complex Ti^2^L_2_ adopted a distorted octahedral configuration with each titanium coordinated with an oxygen, a nitrogen, a sulfur and three chlorine atoms. The O1–C9 and O2–C22 bond lengths were 1.338(6) and 1.322(6) Å, respectively, which were appreciably longer than the corresponding O1–C12 and O2–C25 bonds in the ligand. Compared with the C11–C12 and C24–C25 bonds in ligand, the corresponding C9–C8 and C22–C21 bond lengths (1.351(7), 1.367(7) Å) were significantly shorter and clearly showed some double bond characteristic. Furthermore, compared with the C–C and C–N bonds in ligand, the corresponding C7–C8 and C20–C21 bonds were elongated, and the C7–N1 and C20–N2 were shortened, all of which demonstrated that the ligand has converted to β-imino enol form after coordinating with titanium.

The bond angle sum of O1–Ti1–N1 (84.26°), N1–Ti1–S1 (77.11°), S1–Ti1–Cl3 (93.71°) and Cl3–Ti1–O1 (104.94°) is nearly 360°, so the N1, O1, S1, Cl3 and Ti1 are almost coplanar. The same is true for N2, O2, S2, Cl6 and Ti2. The three chlorine atoms on Ti1 are in a *mer* disposition with the bond angles of Cl1–Ti–Cl3, Cl2–Ti–Cl3 and Cl1–Ti–Cl2 of 93.22(6), 92.34(6) and 168.04(7)°, respectively, which is favorable for the olefin coordination and insertion. The Ti1–O1, Ti1–N1 and Ti1–S1 distances and the average Ti–Cl distance are close to the mononuclear β-carbonylenamine-derived [O^−^NS]TiCl_3_ complexes reported by Tang's group. The bond angle sum of C1–S1–C36 (103.7(3)°), C1–S1–Ti1 (95.87(17)°), and C36–S1–Ti1 (112.2(2)°) is 311.77°, suggesting that the S atom in Ti^2^L_2_ is sp^3^-hybridized. The dihedral angel between N1–C7–C8–C9–O1 and N1–Ti1–O1 or the C14–C15–C16–C17–C18–C19 ring are 32.98° and 42.35°, and the dihedral angel between N2–C20–C21–C22–O2 and N2–Ti2–O2 or the C14–C15–C16–C17–C18–C19 ring are 29.47° and 59.08°, respectively.

### 2.2. Ethylene polymerization

We investigated the catalytic performances of binuclear complexes Ti^2^L_1_–Ti^2^L_5_ towards ethylene polymerization under activation of MMAO, with the mononuclear analogue TiL_6_ for comparison, and the results were listed in Table 3.

**Table 3:** The results of ethylene polymerization catalyzed by binuclear Ti complexes^a^

Entry	Cat.	Al/Ti	Temp (°C)	PE (g)	Act.^b^	*M*_w_ c	*M*_w_/*M*_n_
1	Ti^2^L_2_	1000 : 1	30	0.3200	0.96		
2	Ti^2^L_2_	1000 : 1	50	0.5608	1.68	3.62	3.65
3	Ti^2^L_2_	1000 : 1	70	0.3029	0.91		
4	Ti^2^L_2_	500 : 1	50	0.3578	1.07		
5	Ti^2^L_2_	1500 : 1	50	0.3813	1.14		
6	Ti^2^L_2_	2000 : 1	50	0.3342	1.00		
7	Ti^2^L_1_	1000 : 1	50	0.4421	1.33	4.53	2.82
8	Ti^2^L_3_	1000 : 1	50	0.3634	1.09	4.95	4.30
9	Ti^2^L_4_	1000 : 1	50	0.2736	0.82	4.47	4.08
10	Ti^2^L_5_	1000 : 1	50	0.6451	1.94	14.82	2.65
11^d^	TiL_6_	1000 : 1	50	0.5159	1.55	5.24	2.59
12^d^	TiL_6_	1000 : 1	70	0.1415	0.42		

a Toluene 30 ml, 2 μmol of catalyst, 1 atm ethylene pressure, reaction time 5 min.

b Activity, 10^6^ g mol (Ti)^−1^ h^−1^ atm^−1^.

c 10^4^ g mol^−1^, determined by GPC using polystyrene standard.

d 4 μmol of catalyst.

In general, these binuclear complexes exhibited very high activity (over 10^6^ g mol^−1^ h^−1^ atm^−1^) under suitable conditions, producing typical high-density polyethylene. The polymerization conditions such as reaction temperatures and Al/Ti molar ratios exerted great influence upon catalytic activity and polymer properties.

Firstly, we used Ti^2^L_2_ as catalyst precursor and explored the influence of polymerization temperature at 1 atm ethylene pressure with Al/Ti ratio fixed at 1000. When the reaction temperature was increased from 30 to 70 °C, the activity increased gradually to a maximum at 50 °C and then decreased slightly. The highest activity reached 1.68 × 10^6^ g mol (Ti)^−1^ h^−1^ atm^−1^ at 50 °C (entry 2, Table 3), which was similar to that catalyzed by the mononuclear analogue TiL_6_/MMAO (1.55 × 10^6^ g mol (Ti)^−1^ h^−1^ atm^−1^, entry 10 in Table 3). However, the binuclear complex appeared more stable at elevated temperature compared with the mononuclear TiL_6_. At 70 °C, the activity of Ti^2^L_2_/MMAO was still of 9.1 × 10^5^ g mol (Ti)^−1^ h^−1^ atm^−1^, which was more than twice that of TiL_6_/MMAO at the same temperature.

Both bi- and mono-nuclear titanium catalysts catalyzed ethylene polymerization to produce polyethylene with over 10^4^ g mol^−1^ of molecular weight (*M*_w_). The molecular weight distribution (*M*_w_/*M*_n_) of polyethylene produced by TiL_6_/MMAO was only 2.59, which was typical of single active center; however the polymer obtained with Ti^2^L_2_/MMAO exhibited much wider polydispersity (3.65), indicating that two active centers may have formed, consistent with the asymmetrical crystal structure of the binuclear complex.

The catalytic activity of the binuclear catalyst was less sensitive to the Al/Ti molar ratio. The catalyst exhibited high activity of over 10^6^ g mol (Ti)^−1^ h^−1^ atm^−1^ even at a low Al/Ti ratio of 500, and with Al/Ti ratio increased from 500 to 2000, the activity increased slightly to a maximum at an Al/Ti ratio of 1000 and then slowly decreased.

The catalytic performances of the binuclear complexes bearing different alkylthio and phenylthio sidearms were also compared. The steric hindrance of substituents on sulfur atom influenced both the catalytic activity and molecular weight of the resulting polyethylene. Take Ti^2^L_2_ and Ti^2^L_3_ for example (entry 2 *vs.* 8, Table 3), as the sidearm *n*-propylthio changed to bulkier *iso*-propylthio, the catalytic activity decreased from 1.68 to 1.09 × 10^6^ g mol (Ti)^−1^ h^−1^ atm^−1^, while the molecular weight increased from 3.62 to 4.95 × 10^4^ g mol^−1^. The complex Ti^2^L_5_ which bears the bulkier phenylthio sidearm produced polyethylene with still-higher molecular weight than those with the alkylthio sidearms (entry 10 *vs.* 2, 7–9, Table 3). Similar influences of steric hindrance have also been observed in mononuclear titanium complexes.^9*b*^

**Fig. 4:**
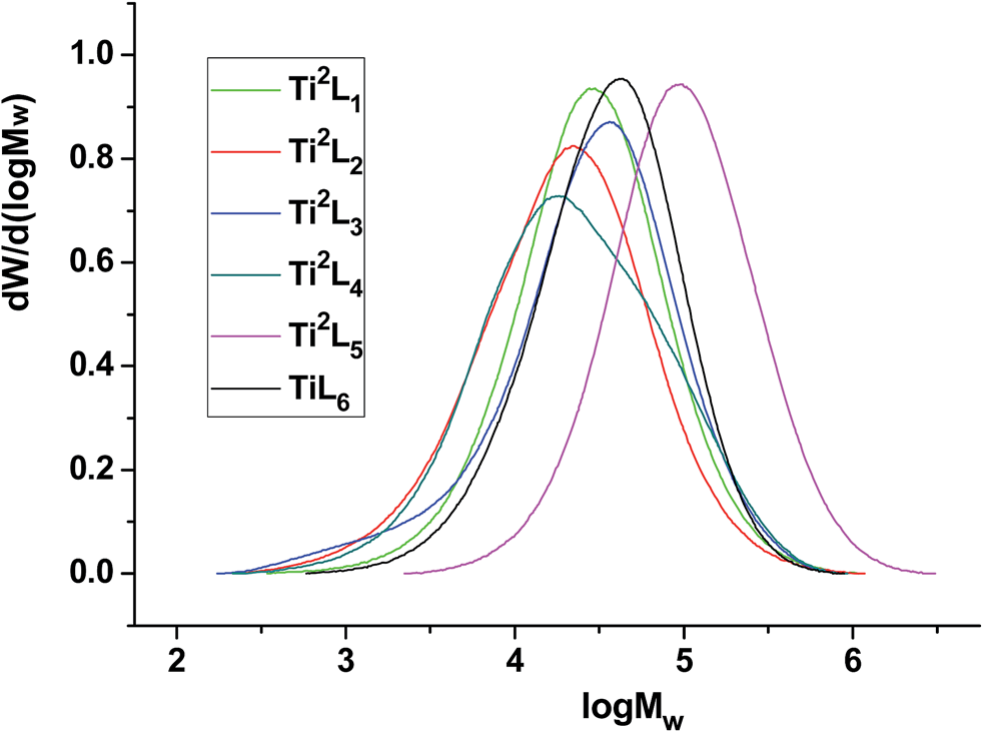
GPC curves for the PE samples obtained with bi- and mono-nuclear Ti complexes.

The influence of substituents was also investigated by varying the length of the linear alkylthio sidearms. Unlike the mononuclear analogues reported by Tang's group^9*c*^ and the methylene-bridged salicylaldiminato binuclear titanium complexes reported by us^15*a*^ previously, the catalytic activity decreased from 1.68 to 0.82 × 10^6^ g mol (Ti)^−1^ h^−1^ atm^−1^ (entry 2 *vs.* 9, Table 3) when the substituent on sulfur atom was changed from *n*-propyl group to *n*-octyl group. However, replacement of *n*-propyl group with methyl group also decreased the activity slightly, due probably to the weaker solubility of Ti^2^L_1_ (entry 2 *vs.* 7, Table 3). With the increase of the alkyl chain length of the side group on sulfur atom, the molecular weight distribution of obtained PE increased gradually, while the molecular weight remained almost unchanged. The GPC curves for the PE samples were shown in Fig. 4.

### 2.3. Ethylene copolymerization with α-olefins

We also explored the catalytic behaviors of these binuclear complexes towards ethylene copolymerization with α-olefins, and the results were shown in Table 4.

**Table 4:** Copolymerization of ethylene and α-olefins catalyzed by binuclear Ti complexes^a^

Entry	Cat.	Comonomer (mmol)	Polymer (g)	Act. b	*T*_m_ c (°C)	*M*_w_ d	*M*_w_/*M*_n_	Incorp^e^ (mol%)
1	Ti^2^L_2_	C6(6)	0.6807	2.04	107.0			5.1
2	Ti^2^L_2_	C6(12)	1.4062	4.22	94.6	8.42	3.89	11.3
3	Ti^2^L_2_	C6(24)	1.8634	5.59	84.5			18.7
4	Ti^2^L_2_	C6(36)	1.4110	4.23	—			19.1
5	Ti^2^L_1_	C6(12)	0.6548	1.96	99.2	3.42	3.20	18.3
6	Ti^2^L_3_	C6(12)	1.2367	3.71	104.2	15.12	2.82	9.6
7	Ti^2^L_4_	C6(12)	0.9662	2.90	88.9	14.51	2.75	7.7
8	Ti^2^L_5_	C6(12)	1.0759	3.23	93.3	12.93	2.39	6.3
9^f^	TiL_6_	C6(12)	1.8898	5.67	106.7	3.36	3.04	17.2
10	Ti^2^L_2_	C8(12)	1.5343	4.60	97.1			5.5
11	Ti^2^L_2_	C10(12)	1.7985	5.40	96.9			8.4

a Toluene 30 ml, 2 μmol of catalyst, 1 atm ethylene pressure, 1000 Al/Ti molar ratio, polymerization temperature 30 °C, reaction time 5 min.

b Activity, 10^6^ g mol (Ti)^−1^ h^−1^ atm^−1^.

c Melting temperature determined by DSC.

d 10^4^ g mol^−1^, determined by GPC using polystyrene standard.

e Determined by high temperature ^13^C NMR.

f 4 μmol of catalyst.

All of these complexes showed extremely high activity for the copolymerization of ethylene and α-olefins, which were 2–5 times higher than the homopolymerization activity (Table 3). The products were branched polyethylene as revealed by their much reduced melting points and the high temperature ^13^C NMR spectra. The 1-hexene incorporation ratio in the copolymer could be flexibly tuned by the initial feed of α-olefin commoners and catalyst structures. It should be noted that these bis-β-carbonylenamine-derived binuclear titanium complexes showed much higher copolymerization activity and α-olefin incorporation ratio compared with the methylene-bridged bis-salicylaldiminato binuclear titanium complexes reported by us before under similar conditions.^15*a*^

The influences of 1-hexene feeds upon catalytic performances were investigated with Ti^2^L_2_/MMAO as a representative. As the feed of 1-hexene was increased from 6 to 36 mmol, the 1-hexene incorporation ratio increased sharply from 5.1 to 19.1 mol% (calculated from the ^13^C NMR spectra, entry 1–4, Table 4), while the copolymerization activity increased from 2.04 × 10^6^ g mol (Ti)^−1^ h^−1^ atm^−1^ to a maximum of 5.59 × 10^6^ g mol (Ti)^−1^ h^−1^ atm^−1^ at 24 mmol of 1-hexene, and then decreased slightly to 4.23 × 10^6^ g mol (Ti)^−1^ h^−1^ atm^−1^ at 36 mmol. It appeared that within a certain range the activity of the binuclear Ti complex increased apparently with the increase of 1-hexene concentration, showing positive “comonomer effect”.

The structure of binuclear titanium complexes also affected their catalytic performances for ethylene/1-hexene copolymerization. Under the same conditions, the increase of steric hindrance of the substituents on sulfur atom reduced the copolymerization activity and 1-hexene incorporation ratio, but increased the molecular weight of obtained copolymers (entry 2 *vs.* 6, *n*-propyl *vs. iso*-propyl, entry 2 *vs.* 7, *n*-propyl *vs. n*-octyl, Table 4). Furthermore, replacement of *n*-propyl group with smaller sized methyl group enhanced significantly the 1-hexene incorporation ratio from 11.3 to 18.3 mol% and decreased the molecular weight from 8.42 to 3.42 × 10^4^ g mol^−1^ (entry 2 *vs.* 5, Table 4). However, replacement of alkyl group with phenyl group on sulfur atom lowered the 1-hexene incorporation ratio (entry 2, 5–7 *vs.* 8, Table 4), which was in good accord with the salicylaldiminato mononuclear titanium complexes reported by Tang.^9^

**Fig. 5:**
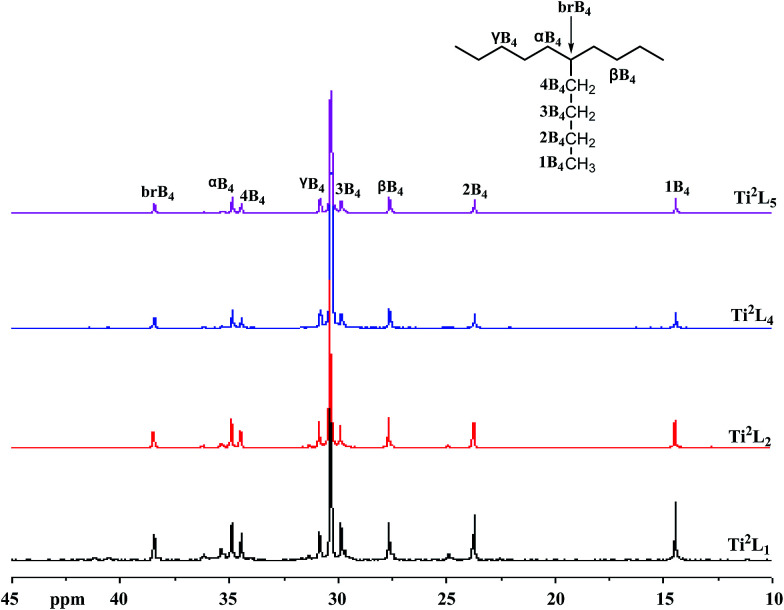
^13^C NMR spectra of PE samples from entries 2, 5, 7 and 8 in Table 4.

**Fig. 6:**
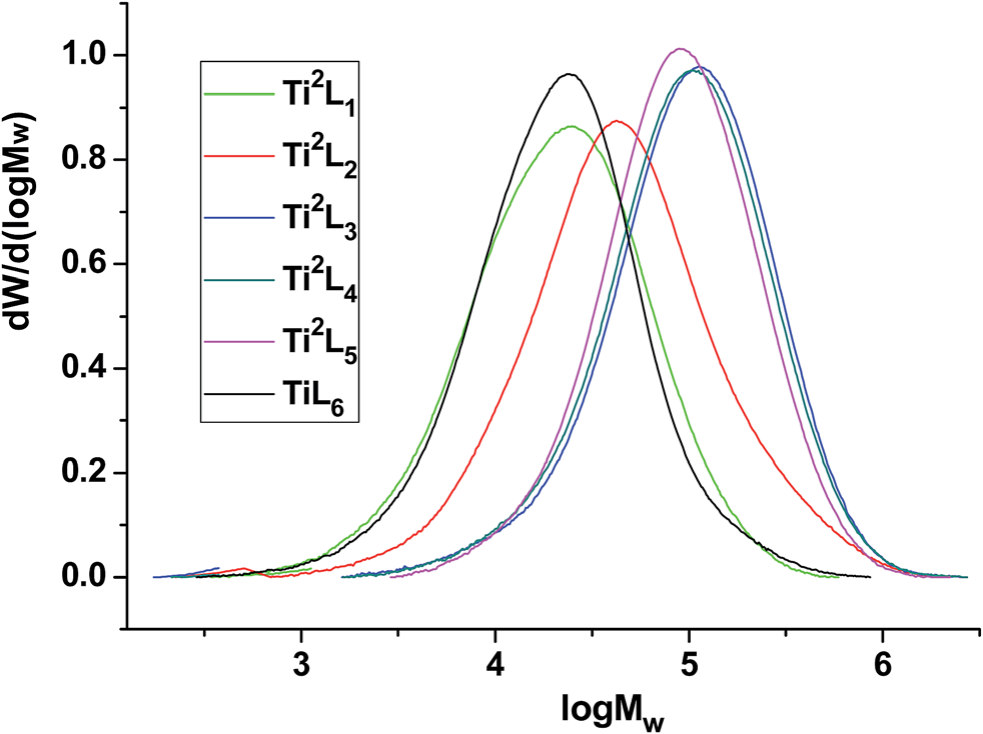
GPC curves of the ethylene/1-hexene copolymers obtained with bi- and mono-nuclear Ti complexes.

The high temperature ^13^C NMR spectra of copolymers produced by Ti^2^L_1_, Ti^2^L_2_, Ti^2^L_4_ and Ti^2^L_5_ were shown in Fig. 5, with the corresponding carbon units marked for different peaks. Variation of branch density can be clearly observed, as demonstrated by the relative peak heights. The GPC curves for the ethylene/1-hexene copolymers obtained with bi- and mono-nuclear Ti complexes were shown in Fig. 6.

Under the same conditions, complex Ti^2^L_2_ demonstrated lower copolymerization activity and 1-hexene incorporation ratio than those of its mononuclear counterpart TiL_6_ (entry 2 *vs.* 9, Table 4), probably due to the steric and electronic effects of the altered coordination environment. This type of binuclear titanium complexes showed negligible bimetallic cooperative effects due to the far-separation of the two titanium centers.

The binuclear titanium complex Ti^2^L_2_ could also efficiently catalyze ethylene copolymerization with 1-octene or 1-decene, with higher activity but lower comonomer incorporation ratio (entry 10 and 11, Table 4) compared with ethylene/1-hexene copolymerization.

## 3 Conclusions

A series of phenyl-bridged bis-β-carbonylenamine [ONS^R^] (R = alkyl or phenyl) tridentate ligands L_1_–L_5_ and their binuclear titanium complexes Ti^2^L_1_–Ti^2^L_5_ were synthesized and characterized. The molecular structures of ligand L_2_ (R = ^*n*^Pr) and its corresponding Ti complex Ti^2^L_2_ as studied by single-crystal X-ray diffraction revealed that each titanium coordinated with an oxygen, a nitrogen, a sulfur and three chlorine atoms to form a distorted octahedral configuration. Furthermore, the alkylthio or phenylthio anilines reacted with the carbonyl groups adjacent to phenylene group, resulting in isolated and relatively independent titanium centers in the complex. Compared with the mononuclear analogue TiL_6_, these complexes exhibited better thermal stability for ethylene polymerization and produced PE with higher molecular weight and wider polydispersity, suggesting that two active centers were formed. The molecular weight and α-olefin incorporation ratio can be flexibly tuned by the catalyst structure. The complex Ti^2^L_5_ which bears phenylthio sidearm exhibited higher activity towards ethylene polymerization and produced polyethylene with much higher molecular weight compared with the complexes bearing alkylthio sidearms, but resulted in lower 1-hexene incorporation ratio. Meanwhile, the 1-hexene incorporation ratio could also be tuned by the initial feed of the α-olefin commoner. However, this type of binuclear titanium complexes showed weak or negligible bimetallic cooperative effects due to the far separation of the titanium centers.

## 4 Experimental section

### 4.1. General procedures

All manipulations involving air- and/or moisture-sensitive compounds were performed under dry nitrogen using standard Schlenk-line and glovebox. Toluene and hexane were purified by distillation over sodium/benzophenone ketyl, while CH_2_Cl_2_ was refluxed over CaH_2_. Gases and other solvents were purified by standard techniques. Modified methylaluminoxane (MMAO) was purchased from Akzo Chemical as a 7 wt% solution in heptane. All other chemical reagents were used as received unless noted otherwise.

^1^H and ^13^C NMR spectra of ligands and complexes were recorded on a Bruker Avance III 400 MHz spectrometer with tetramethylsilane as an internal standard. Elemental analyses were carried out using Vario EL 111. ^13^C NMR spectra of polymers were obtained on a Varian XL 300 MHz spectrometer at 120 °C with *o*-C_6_D_4_Cl_2_ as the solvent. IR spectra were collected with a Nicolet Nexus 470 Fourier transform infrared (FTIR) spectrometer. DSC measurements were performed on a Netzsch DSC200 F3 instrument at a heating rate of 10 °C min^−1^ from 20 to 160 °C, with the melting points obtained from the endothermic peak of the second heating scan. The *M*_n_ and *M*_w_/*M*_n_ of the polymers were determined at 150 °C with a Viscotek 350A HT-GPC System using a polystyrene calibration. 1,2,4-Trichlorobenzene was employed as the solvent at a flow rate of 1.0 mL min^−1^.

### 4.2. Synthesis of the ligands and binuclear titanium complexes

#### 4.2.1 1,1′-(1,3-Phenylene)-bis-(4,4-dimethylpentane-1,3-dione) (2)

2.9 g of dimethyl isophthalate (1), 3.7 g of pinacolone, 4.6 g of sodium amide and 40 ml of absolute ether dried by sodium were added to a 100 ml 3-neck flask, and stirred at 0 °C for 2 h, and then warmed to RT to react for another 2 h. The reaction was quenched by 50 ml ice water, then an orange yellow solid was produced, which was washed with ice ethanol for three times to obtain 3.2 g of off-white product with 65% yield. ^1^H NMR (400 MHz, CDCl_3_): *δ* 8.40 (s, 1H), 8.04 (s, 2H), 7.56 (d, *J* = 7.8 Hz, 1H), 6.36 (s, 2H), 1.28 (s, 18H). Anal. calcd for C_20_H_26_O_4_: C, 72.70; H, 7.93%. Found: C, 72.32; H, 7.96%.

#### 4.2.2 4,4-Dimethyl-1-phenylpentane-1,3-dione (4)

4 was synthesized with a similar procedure as for 2. ^1^H NMR (400 MHz, CDCl_3_): *δ* 7.92 (s, 2H), 7.52 (s, 1H), 7.46 (d, *J* = 6.2 Hz, 2H), 6.33 (s, 1H), 1.28 (s, 9H). Anal. calcd for C_13_H_16_O_2_: C, C, 76.44; H, 7.90%. Found: C, 76.85; H, 7.46%.

#### 4.2.3 Ligands L_1_–L_6_

##### L_1_

To a 100 ml 3-neck flask were added 0.6837 g of 2, 0.6791 g of methylthio aniline, 40 ml of toluene, and 0.1 g of *p*-toluene sulfonic acid (TsOH) as catalyst, and heated to reflux for 24 h. The mixture was then vacuum dried, and recrystallized with ethanol to afford L_1_ as a yellow solid with 78% yield. ^1^H NMR (400 MHz, CDCl_3_): *δ* 12.05 (s, 1H), 7.30 (s, 1H), 7.25 (s, 1H), 7.23 (d, *J* = 5.0 Hz, 1H), 7.21–7.13 (m, 1H), 7.00 (t, *J* = 7.7 Hz, 1H), 6.79 (t, *J* = 7.6 Hz, 1H), 6.19 (d, *J* = 7.7 Hz, 1H), 5.45 (s, 1H), 2.51 (s, 3H), 1.24 (d, *J* = 21.4 Hz, 9H). ^13^C NMR (101 MHz, CDCl_3_): *δ* 206.30 (*C*=O), 158.87 (Ar-NH–*C*), 137.91, 136.55, 132.38, 129.14, 128.52, 127.94, 126.51, 125.14, 124.75, 124.71 (Ar-*C*), 97.14, 42.56, 27.60, 15.53. Anal. calcd for C_34_H_40_N_2_O_2_S_2_: C, 71.29; H, 7.04; N, 4.89%. Found: C, 71.38; H, 6.92; N, 4.75%. IR (KBr, cm^−1^): 3452, 3070, 2964, 1608, 1501, 1475, 1441, 1325, 1219, 1122, 1087, 751.

Ligands L_2_–L_6_ were synthesized with a similar procedure as for L_1_.

##### L_2_

Yield: 80%. ^1^H NMR (400 MHz, CDCl_3_): *δ* 12.06 (s, 2H), 7.31 (s, 1H), 7.30 (s, 2H), 7.24 (d, *J* = 7.1 Hz, 2H), 7.19 (d, *J* = 8.6 Hz, 1H), 6.95 (t, *J* = 7.3 Hz, 2H), 6.81 (t, *J* = 7.3 Hz, 2H), 6.20 (d, *J* = 7.9 Hz, 2H), 2.93 (t, *J* = 7.3 Hz, 4H), 1.81–1.67 (m, 4H), 1.21 (s, 18H), 1.07 (t, *J* = 7.4 Hz, 6H). ^13^C NMR (101 MHz, CDCl_3_): *δ* 206.13 (*C*=O), 158.43 (Ar-NH–*C*), 139.19, 136.73, 130.21, 129.50, 129.11, 126.74, 125.82, 125.54, 124.97, 124.31 (Ar-*C*), 97.35, 42.56, 34.97, 27.61, 22.50, 13.62. Anal. calcd for C_38_H_48_N_2_O_2_S_2_: C, 72.57; H, 7.69; N, 4.45%. Found: C, 72.83; H, 7.88; N, 4.16%. IR (KBr, cm^−1^): 3446, 3063, 2959, 2868, 1615, 1589, 1571, 1533, 1497, 1474, 1451, 1325, 1286, 1123, 1088, 751.

##### L_3_

Yield: 68%. ^1^H NMR (400 MHz, CDCl_3_): *δ* 12.14 (s, 1H), 7.39 (d, *J* = 6.6 Hz, 1H), 7.29 (s, 1H), 7.25 (s, 1H), 6.90 (d, *J* = 21.1 Hz, 2H), 6.22 (s, 1H), 5.45 (s, 1H), 3.48 (s, 1H), 1.38 (s, 5H), 1.21 (s, 9H). ^13^C NMR (101 MHz, CDCl_3_): *δ* 203.30 (*C*=O), 183.66 (Ar-NH–*C*), 150.68, 138.85, 137.37, 136.14, 131.15, 130.33, 128.94, 125.87, 125.48, 117.36 (Ar-*C*), 92.40, 39.97, 36.12, 27.40, 23.02. Anal. calcd for C_38_H_48_N_2_O_2_S_2_: C, 72.57; H, 7.69; N, 4.45%. Found: C, 72.29; H, 7.35; N, 4.68%. IR (KBr, cm^−1^): 3451, 3061, 2966, 1623, 1386, 1316, 1189, 1088, 569, 461.

##### L_4_

Yield: 78%. ^1^H NMR (400 MHz, CDCl_3_): *δ* 12.08 (d, *J* = 20.4 Hz, 2H), 7.30 (d, *J* = 4.1 Hz, 2H), 7.29 (s, 1H), 7.27–7.22 (m, 2H), 7.22–7.14 (m, 1H), 6.95 (t, *J* = 7.7 Hz, 2H), 6.80 (t, *J* = 7.5 Hz, 2H), 6.20 (d, *J* = 8.2 Hz, 2H), 5.44 (s, 2H), 2.94 (t, *J* = 7.4 Hz, 4H), 1.77–1.67 (m, 4H), 1.46 (dd, *J* = 13.4, 6.5 Hz, 4H), 1.29 (dd, *J* = 9.8, 4.0 Hz, 16H), 1.21 (s, 18H), 0.89 (t, *J* = 6.5 Hz, 6H). ^13^C NMR (101 MHz, CDCl_3_): *δ* 206.02 (*C*=O), 158.39 (Ar-NH–*C*), 139.26, 136.78, 130.38, 129.56, 129.09, 128.47, 127.93, 125.53, 124.93, 124.28 (Ar-*C*), 97.37, 42.54, 39.24, 33.16, 31.82, 29.19, 28.97, 28.70, 27.61, 22.65, 14.10. Anal. calcd for C_48_H_68_N_2_O_2_S_2_: C, 74.95; H, 8.91; N, 3.64%. Found: C, 74.59; H, 8.62; N, 3.91%. IR (KBr, cm^−1^): 3448, 3061, 2926, 2858, 1608, 1564, 1466, 1315, 1220, 1188, 1100, 788.

##### L_5_

Yield: 83%. ^1^H NMR (400 MHz, CDCl_3_): *δ* 12.29 (s, 2H), 7.43–6.83 (m, 22H), 6.20 (d, *J* = 7.7 Hz, 2H), 1.21 (s, 18H). ^13^C NMR (101 MHz, CDCl_3_): *δ* 206.11 (*C*=O), 158.26 (Ar-NH–*C*), 139.83, 136.59, 134.33, 132.50, 132.19, 129.48, 129.19, 128.94, 128.44, 127.73, 127.54, 127.16, 125.01, 124.37 (Ar-*C*), 97.24, 42.53, 27.61. Anal. calcd for C_44_H_44_N_2_O_2_S_2_: C, 75.83; H, 6.36; N, 4.02%. Found: C, 75.62; H, 6.53; N, 4.34%. IR (KBr, cm^−1^): 3447, 3059, 2964, 2865, 2360, 2342, 1616, 1571, 1558, 1497, 1475, 1439, 1289, 1120, 1089, 1023, 792, 750.

##### L_6_

Yield: 81%. ^1^H NMR (400 MHz, CDCl_3_): *δ* 12.22 (s, 1H), 7.30 (d, *J* = 11.2 Hz, 6H), 6.85 (d, *J* = 47.5 Hz, 2H), 6.34 (s, 1H), 5.66 (s, 1H), 2.95 (d, *J* = 4.7 Hz, 2H), 1.75 (s, 2H), 1.26 (s, 9H), 1.09 (d, *J* = 8.5 Hz, 3H). ^13^C NMR (101 MHz, CDCl_3_): *δ* 205.91 (*C*=O), 159.32 (Ar-NH–*C*), 139.77, 136.51, 132.89, 130.15, 129.56, 129.41, 128.44, 128.35, 128.14, 125.88, 124.48, 123.90 (Ar-*C*), 97.37, 52.07, 35.31, 27.71, 22.58, 13.56. Anal. calcd for C_22_H_27_NOS: C, 74.75; H, 7.70; N, 3.96%. Found: C, 74.39; H, 7.57; N, 3.73%. IR (KBr, cm^−1^): 3449, 2963, 1617, 1562, 1507, 1387, 1299, 1218, 1186, 1086, 802, 766.

### 4.3. Binuclear titanium complexes (Ti^2^L_1_–Ti^2^L_4_)

#### 4.3.1 Ti^2^L_1_

0.573 g (1 mmol) of ligand L_1_ was added to a 100 ml Schlenk flask, dissolved in 15 ml of CH_2_Cl_2_. A solution of TiCl_4_ (0.28 ml, 2.6 mmol) in CH_2_Cl_2_ (15 ml) was added to another 100 ml Schlenk flask. The ligand solution was then slowly added to the TiCl_4_ solution at −78 °C. After 5 hours, the mixture was slowly warmed to RT, then heated to 35 °C for 24 h under stirring. The solvent was vacuum dried to afford Ti^2^L_1_ as a red brown solid. Pure product was obtained by diffusing *n*-hexane into dichloromethane solution of Ti^2^L_1_ with 67% yield. ^1^H NMR (400 MHz, CDCl_3_): *δ* 7.50 (s, 1H), 7.34 (s, 1H), 7.14 (t, *J* = 7.3 Hz, 1H), 6.95 (s, 2H), 6.21 (s, 1H), 5.94 (s, 1H), 3.15 (d, *J* = 28.7 Hz, 3H), 1.31 (s, 9H). ^13^C NMR (101 MHz, CDCl_3_): *δ* 210.41 (=*C*–O), 172.20 (Ar-N=*C*), 166.35, 151.34, 130.31, 130.02, 128.90, 128.08, 128.04, 125.46, 117.14, 111.60 (Ar-*C*), 92.59, 39.53, 27.79, 14.11. Anal. calcd for C_34_H_38_Cl_6_N_2_O_2_S_2_Ti_2_: C, 46.45; H, 4.36; N, 3.19%. Found: C, 46.82; H, 4.73; N, 3.31%. IR (KBr, cm^−1^): 3070, 2962, 2931, 2875, 1706, 1609, 1558, 1458, 1318, 1275, 1211, 1125, 1091, 765.

Complexes Ti^2^L_2_–Ti^2^L_5_ were prepared using the same procedure as for Ti^2^L_1_.

#### 4.3.2 Ti^2^L_2_

Yield: 75%. ^1^H NMR (400 MHz, CDCl_3_): *δ* 7.57 (d, *J* = 7.0 Hz, 1H), 7.33 (d, *J* = 6.7 Hz, 1H), 7.19 (dd, *J* = 7.7, 3.1 Hz, 1H), 7.13 (t, *J* = 7.6 Hz, 1H), 6.99 (s, 1H), 6.24 (d, *J* = 20.7 Hz, 1H), 6.01 (s, 1H), 3.52 (s, 2H), 2.10 (dd, *J* = 14.8, 7.3 Hz, 2H), 1.32 (s, 9H), 1.23 (t, *J* = 7.3 Hz, 3H). ^13^C NMR (101 MHz, CDCl_3_): *δ* 204.32 (=*C*–O), 165.78 (Ar-N=*C*), 149.71, 141.36, 139.37, 136.75, 129.63, 126.57, 125.01, 117.34, 115.96, 113.45, 113.20, 111.53 (Ar-*C*), 98.55, 31.58, 27.55, 22.64, 14.10, 13.56. Anal. calcd for C_38_H_46_Cl_6_N_2_O_2_S_2_Ti_2_: C, 48.80; H, 4.96; N, 3.00%. Found: C, 49.25; H, 4.64; N, 3.43%. IR (KBr, cm^−1^): 2965, 1620, 1563, 1460, 1289, 1208, 1091, 838, 756.

#### 4.3.3 Ti^2^L_3_

Yield: 80%. ^1^H NMR (400 MHz, CDCl_3_) *δ* 7.56 (d, *J* = 7.9 Hz, 2H), 7.15 (dd, *J* = 35.8, 18.6 Hz, 3H), 6.40 (s, 1H), 6.09 (s, 1H), 4.14 (s, 1H), 1.63 (s, 6H), 1.32 (s, 9H). ^13^C NMR (101 MHz, CDCl_3_): *δ* 199.20 (=*C*–O), 181.31 (Ar-N=*C*), 160.32, 139.43, 136.34, 131.05, 129.67, 129.26, 128.02, 124.41, 112.45, 103.22 (Ar-*C*), 93.69, 31.58, 27.82, 27.54, 23.08. Anal. calcd for C_38_H_46_Cl_6_N_2_O_2_S_2_Ti_2_: C, 48.80; H, 4.96; N, 3.00%. Found: C, 48.51; H, 5.30; N, 2.76%. IR (KBr, cm^−1^): 2964, 1617, 1558, 1497, 1475, 1290, 1120, 1088, 884, 791, 755.

#### 4.3.4 Ti^2^L_4_

Yield: 74%. ^1^H NMR (400 MHz, CDCl_3_): *δ* 7.57 (d, *J* = 7.7 Hz, 2H), 7.13 (t, *J* = 6.9 Hz, 1H), 6.99 (s, 2H), 6.27 (s, 1H), 6.01 (s, 1H), 3.54 (s, 2H), 2.10–2.01 (m, 2H), 1.63–1.57 (m, 2H), 1.43 (d, *J* = 9.2 Hz, 3H), 1.37 (d, *J* = 8.0 Hz, 9H), 1.28 (s, 8H). ^13^C NMR (101 MHz, CDCl_3_): *δ* 206.10 (=*C*–O), 157.75 (Ar-N=*C*), 139.10, 136.71, 130.39, 129.34, 129.12, 127.91, 125.47, 124.96, 124.32, 111.80 (Ar-*C*), 97.36, 42.57, 33.04, 31.83, 31.61, 29.21, 29.14, 27.83, 27.61, 22.67, 14.16. Anal. calcd for C_48_H_66_Cl_6_N_2_O_2_S_2_Ti_2_: C, 53.60; H, 6.19; N, 2.60%. Found: C, 53.39; H, 6.37; N, 2.81%. IR (KBr, cm^−1^): 2956, 2925, 2854, 1617, 1574, 1560, 1483, 1461, 1264, 1146, 891, 762.

#### 4.3.5 Ti^2^L_5_

Yield: 68%. ^1^H NMR (400 MHz, CDCl_3_): *δ* 7.50–7.28 (m, 22H), 5.83 (s, 2H), 0.90 (t, *J* = 6.5 Hz, 18H). ^13^C NMR (101 MHz, CDCl_3_) *δ* 203.48 (=*C*–O), 173.45 (Ar-N=*C*), 156.12, 147.72, 144.86, 139.70, 134.83, 134.21, 132.26, 130.47, 129.63, 129.50, 129.43, 121.37, 109.07, 107.56 (Ar-*C*), 86.99, 31.58, 22.65. Anal. calcd for C_44_H_42_Cl_6_N_2_O_2_S_2_Ti_2_: C, 52.67; H, 4.22; N, 2.79%. Found: C, 52.18; H, 4.86; N, 3.31%. IR (KBr, cm^−1^): 3331, 2963, 1707, 1557, 1496, 1475, 1439, 1347, 1290, 1210, 1122, 1087, 1059, 1023, 793, 750, 691.

### 4.4 Mononuclear titanium complex (TiL_6_)

TiL_6_ was prepared using the same procedure as for Ti^2^L_1_, except that the molar ratio of ligand L_6_ and TiCl_4_ was 1 : 1.2. Yield: 80%. ^1^H NMR (400 MHz, CDCl_3_) *δ* 7.53 (d, *J* = 7.7 Hz, 1H), 7.39 (d, *J* = 7.4 Hz, 1H), 7.34 (t, *J* = 7.3 Hz, 2H), 7.21 (d, *J* = 6.0 Hz, 2H), 7.06 (t, *J* = 7.1 Hz, 1H), 6.92 (t, *J* = 7.6 Hz, 1H), 6.45 (d, *J* = 8.2 Hz, 1H), 6.26 (s, 1H), 3.50 (s, 2H), 2.06 (dd, *J* = 14.9, 7.5 Hz, 2H), 1.36 (s, 9H), 1.21 (t, *J* = 7.4 Hz, 3H). ^13^C NMR (101 MHz, CDCl_3_): *δ* 190.20 (=*C*–O), 173.64(Ar-N=*C*), 152.70, 138.76, 133.40, 133.36, 130.56, 129.59, 129.15, 128.67, 128.51, 127.33, 125.72, 112.00 (Ar-*C*), 100.13, 47.03, 39.50, 27.81, 21.81, 13.36. Anal. calcd for C_22_H_26_Cl_3_NOSTi: C, 52.15; H, 5.17; N, 2.76%. Found: C, 52.58; H, 5.49; N, 2.36%. IR (KBr, cm^−1^): 2964, 1617, 1559, 1506, 1087, 769.

### 4.5. Crystallographic analysis

Crystal data were collected on a Bruker APEX-II CCD diffractometer with graphite-monochromated Mo Kα radiation (*λ* = 0.71073 Å) at 130 K for Ti^2^L_2_. Crystals were coated in oil and then directly mounted on the diffractometer under a stream of cold nitrogen gas. A total of N reflections were collected by using *ω* scan mode. Corrections were applied for Lorentz and polarization effects as well as absorption using multi-scans (SADABS). All the structures were solved by direct method (SHELXS-97). The remaining non-hydrogen atoms were obtained from the successive difference Fourier maps. All non-H atoms were refined with anisotropic displacement parameters, while the H atoms were constrained to the parent sites, using a riding mode (SHELXTL). Details of the X-ray structure determinations and refinements are provided in Table 1. Other details are shown in the ESI.† CCDC numbers for L_2_ and Ti^2^L_2_ are CCDC 1587147 and 1587146,† respectively.

### 4.6. Ethylene polymerization and copolymerization

A flame-dried Schlenk flask purged with N_2_ was filled with ethylene gas. 30 ml of freshly distilled toluene was added and raised to the reaction temperature for 10 min. MMAO was then injected using a syringe and the mixture was stirred for 5 min. The polymerization was initiated by adding a solution of the titanium complex in toluene with a syringe. After a desired time, the polymerization was quenched with acidified ethanol (100 mL, 8 vol% HCl in ethanol). The precipitated polymer was filtered off, washed with ethanol, then dried under vacuum overnight at 60 °C till a constant weight. For copolymerization, α-olefins (1-hexene, 1-octene or 1-decene) and MMAO were injected in sequence *via* a syringe.

## Conflicts of interest

There are no conflicts to declare.

## Supplementary Material

RA-008-C8RA00071A-s001
